# Analysis of Transcription Factor-Related Regulatory Networks Based on Bioinformatics Analysis and Validation in Hepatocellular Carcinoma

**DOI:** 10.1155/2018/1431396

**Published:** 2018-08-29

**Authors:** Shui Liu, Xiaoxiao Yao, Dan Zhang, Jiyao Sheng, Xin Wen, Qingyu Wang, Gaoyang Chen, Zhaoyan Li, Zhenwu Du, Xuewen Zhang

**Affiliations:** ^1^Department of Hepatobiliary and Pancreatic Surgery, The Second Hospital of Jilin University, Changchun 130041, China; ^2^Jilin Engineering Laboratory for Translational Medicine of Hepatobiliary and Pancreatic Diseases, The Second Hospital of Jilin University, Changchun 130041, China; ^3^The Second Hospital of Jilin University, Changchun 130041, China; ^4^Department of Orthopedics, The Second Hospital of Jilin University, Changchun 130041, China; ^5^Research Center of Second Clinical College, Jilin University, Changchun 130041, China

## Abstract

Hepatocellular carcinoma (HCC) accounts for a significant proportion of liver cancer, which has become the second most common cause of cancer-related mortality worldwide. To investigate the potential mechanisms of invasion and progression of HCC, bioinformatics analysis and validation by qRT-PCR were performed. We found 237 differentially expressed genes (DEGs) including EGR1, FOS, and FOSB, which were three cancer-related transcription factors. Subsequently, we constructed TF-gene network and miRNA-TF-mRNA network based on data obtained from mRNA and miRNA expression profiles for analysis of HCC. We found that 42 key genes from the TF-gene network including EGR1, FOS, and FOSB were most enriched in the p53 signaling pathway. The qRT-PCR data confirmed that mRNA levels of EGR1, FOS, and FOSB all were decreased in HCC tissues. In addition, we confirmed that the mRNA levels of CCNB1, CCNB2, and CHEK1, three key markers of the p53 signaling pathway, were all increased in HCC tissues by bioinformatics analysis and qRT-PCR validation. Therefore, we speculated that miR-181a-5p, which was upregulated in HCC tissues, could regulate FOS and EGR1 to promote the invasion and progression of HCC by p53 signaling pathway. Overall, the study provides support for the possible mechanisms of progression in HCC.

## 1. Introduction

Hepatocellular carcinoma (HCC) is one of the common digestive system malignancies with high mortality, which has become the second most common cause of cancer-related mortality worldwide [1, 2]. There are more than 466,100 estimated new cases and 422,100 estimated death cases every year in China [3]. Although there is extensive research about the therapies for HCC, the specific mechanisms of HCC occurrence and development were not clear. It is significant to investigate the underlying mechanisms of HCC invasion and progression to develop effective diagnostic and therapeutic strategy.

Over the last decades, bioinformatics and microarray technology have been widely used to screen the molecular mechanisms of HCC, which provide powerful research support for the diagnosis and treatment of HCC [4–6]. By bioinformatics analysis and microarray technology, functions of some differentially expressed genes (DEGs) in HCC have been explored, which paved the way for exploring complicated process in the occurrence and development of HCC [5, 7–9]. As an important part of participating in life activities, transcription factors (TFs) have been reported to play an important role in the development of HCC by numerous studies [10, 11]. A work by S. Hua et al. found that ETS1, a cancer-related TF, could through interaction with miR-139-5p inhibit cell proliferation, migration, and invasion in HCC [12]. Another research reported that NF*κ*B/EGR1 signaling pathway induced miR-3928v to promote the progression of HCC [13]. Even so, more research is still needed to explore the specific role of different TFs in the progression of HCC.

During the present study, we analyzed 4 mRNA microarray datasets and 1 miRNA microarray dataset from Gene Expression Omnibus (GEO) to obtain DEGs and differentially expressed miRNAs (DEMs) between HCC tissues and adjacent noncancerous tissues by GEO2R. Subsequently, Gene Ontology (GO), Kyoto Encyclopedia of Genes and Genomes (KEGG) pathway enrichment analysis, and protein-protein interaction (PPI) network analysis were performed to reveal the interaction relationship between DEGs and DEMs and to explore the underlying molecular mechanisms in the carcinogenesis and progression of HCC [14–17]. In addition, we screened differentially expressed TFs in HCC, constructed miRNA-TF-mRNA networks, and proposed a potential miRNA-TF-signaling pathway axis, which identified a systematic exposition of the relevant transcriptional regulation modes associated with invasion and progression of HCC. This study could provide insightful information and the valuable clue for biomarker discovery and novel treatment strategy in HCC.

## 2. Materials and Methods

### 2.1. Microarray Data Information and DEGs Identification

**Figure 1:**
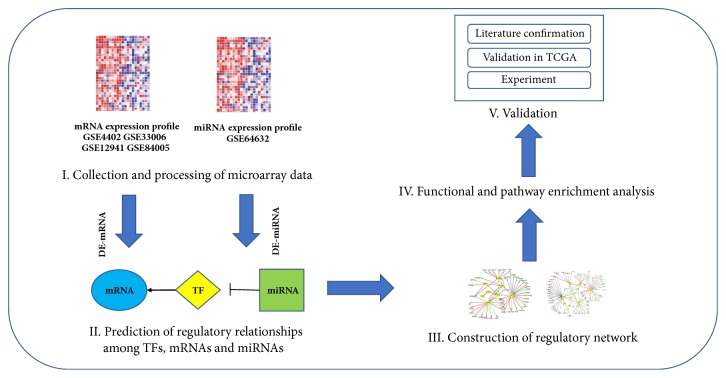
Process of TF-related regulatory network construction and key genes identification in HCC.

NCBI-GEO is a free database of microarray/gene profile and next-generation sequencing, from which HCC and adjacent nontumor tissue gene expression profile of GSE84402, GSE33006, GSE84005, GSE12941, and GSE64632 were obtained [4, 18, 19, 21]. The DEGs and DEMs between HCC tissues and adjacent nontumor tissues were screened using GEO2R (http://www.ncbi.nlm.nih.gov/geo/geo2r), which was an interactive web tool for identifying DEGs across experimental conditions among two or more datasets in a GEO series. Identification of commonly differentially expressed mRNAs from the four cohort profile data sets (GSE84402, GSE33006, GSE84005, and GSE12941) was performed by FunRich software (available online: http://www.funrich.org/). |logFC| (fold change) >1 and P value <0.05 were considered statistically significant. The pipeline of the whole process of this study was shown in Figure 1.

### 2.2. Construction of Transcription Factor Networks

Transcriptional regulatory element database (TRED) (https://cb.utdallas.edu/cgibin/TRED/tred.cgi?process=home) is a database of transcriptional regulatory elements, which provides 36 cancer-related TFs and corresponding regulatory networks [22]. Transcriptional Regulatory Relationships Unraveled by Sentenced-based Text mining version 2.0 database (TRRUST) (available online: http://www.grnpedia.org/trrust/) is a database based on the literature to reflect the relationship between transcriptional regulation [23]. And the prediction of miRNA-target genes was performed by miRTarBase (http://mirtarbase.mbc.nctu.edu.tw/php/index.php) [24]. According to the regulatory interaction, TF-target network and miRNA-TF-mRNA network were constructed based on gene expression profile, TRRUST version 2, TRED, and miRTarBase by Cytoscape 3.6.0 software.

### 2.3. Functional and Pathway Enrichment Analysis

Omicsbean is an online biological information database that integrates biological data and analysis tools and provides a comprehensive set of functional annotation information of genes and proteins for users to extract biological information. To analyze the function of DEGs, GO and KEGG pathway enrichment analysis were performed using Omicsbean online database (http://www.omicsbean.com:88). P value<0.05 was considered statistically significant.

### 2.4. Validation in TCGA Dataset and Modular Analysis of the Key Genes

Validation of the key genes in the TCGA Dataset was performed by UALCAN (http://ualcan.path.uab.edu/index.html) [25]. We performed Kaplan-Meier plots and boxplots to verify the differential expression of the key genes between primary tumor and normal liver and effect of key genes expression level on LIHC patients' survival. Correlation analysis was performed by Linkedomics (http://www.linkedomics.org) [26].

### 2.5. Validation of the Key Genes by Quantitative Real-Time RT-PCR (qRT-PCR)

A total of 20 HCC patients were recruited for tumor and adjacent nontumor tissues collection from the China-Japan Union Hospital of Jilin University, Changchun, China. This study was approved by the Ethics Committee of the Second Clinical Medical College, Jilin University, and each patient consented in a written informed consent form. The data were analyzed anonymously. All tissues were taken from the surgery room and snap-frozen and stored in liquid nitrogen within 10 min after the resection. The clinicopathological characteristics of 20 HCC patients were shown in Table S1.

Tissue RNA was isolated using Trizol (Invitrogen, CA, USA) and further purified using the MiniBEST Universal RNA Extraction Kit (TaKaRa, China) according to the manufacturer's instructions. RNA concentration was then measured using the NanoDrop 2000 spectrophotometer (Thermo Scientific, MA, USA) with A260/A280 ratio in the range 1.8~2.0 and RNA concentration ranged from 100 ng/*µ*l to 1 *µ*g/*µ*l.

For qRT-PCR analysis, less than 5 *µ*g total RNA including microRNA was reverse transcribed to cDNA with 1st strand cDNA Synthesis Kit (Takara, China) and miRNA First Strand cDNA Synthesis (Sangon, China); the expression of mRNA and microRNA was examined by qRT-PCR with TransStart ® Top Green qPCR SuperMix (TransGen Biotech, China), microRNAs qPCR Kit (Sangon, China), and Applied Biosystems 7500 Fast Real-Time PCR System. The relative expression of mRNA and microRNA was normalized to GAPDH/U6 expression by comparative Ct method (2^−ΔΔCt^, ΔCt =* *Ct_target_-Ct_GAPDH/U6_, and ΔΔCt* *=* *ΔCt_tumor_-ΔCt_non-tumor_). All primers were designed with Primer 7.0 Software; primer sequences for amplification were listed in Table 8.

### 2.6. Statistical Analysis

Data from qRT-PCR were analyzed with GraphPad Prism version 7.0, and differences between the two groups were statistically evaluated by two-tailed Student's t-test with p value <0.05 considered as significant. The Pearson correlation coefficient was used to examine the relationship between key genes.

## 3. Results

### 3.1. Identification and Enrichment Analysis of DEGs in HCC

**Figure 2:**
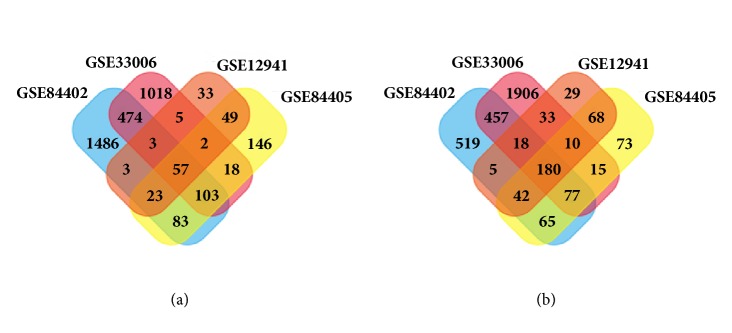
Identification of 237 common DEGs from the four cohort profile data sets. (a) 57 commonly upregulated DEGs. (b) 180 commonly downregulated DEGs. Different color areas represented different datasets. The cross areas meant the commonly changed DEGs. DEGs were identified with classical t-test; statistically significant DEGs were defined with p<0.05 and |logFC|>1 as the cut-off criterion.

HCC and adjacent nontumor tissue gene expression profile of GSE84402, GSE33006, GSE84005, and GSE12941 were obtained from NCBI-GEO. We used p<0.05 and |logFC|>1 as cut-off criterion. After integrated bioinformatical analysis, a total of 237 differentially expressed genes were identified from the four profile datasets, including 57 upregulated genes and 180 downregulated genes (Tables 1–3, Figure 2).

**Figure 3:**
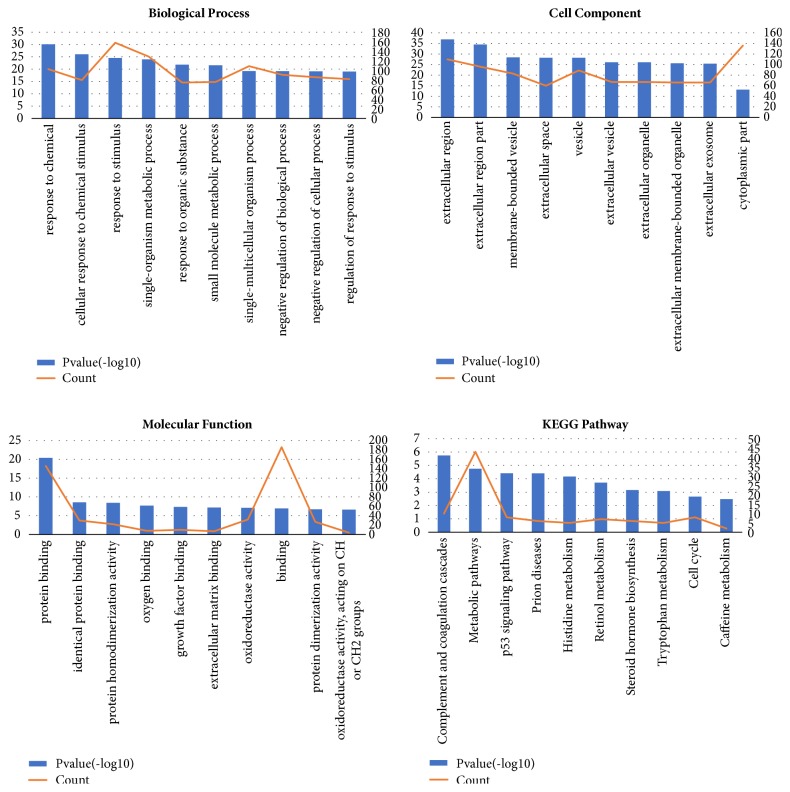
Enrichment analysis of DEGs.

The enrichment analysis of DEGs was performed. As shown in Table 4 and Figure 3, in the biological process group, the DEGs were mainly enriched in response to chemical. In the cellular component group, the DEGs were most enriched in the extracellular region. In the molecular function group, the DEGs were most enriched in protein binding. And the most enriched KEGG pathway was complement and coagulation cascades.

### 3.2. Identification and Functional Analysis of Key Differentially Expressed Genes, Construction of TF-Related Networks in HCC

We intersected 237 common DEGs and curated 36 cancer-related TF families to get three differentially expressed TFs (EGR1, FOS, and FOSB) in HCC (Table 5). We searched the target genes and regulators of the three TFs through TRRUST version 2 database. Based on the results, we found that STAT6, a cancer-related TF, regulated FOS and FOSB, while FOS and EGR1 were coregulated by 2 cancer-related TFs, including BRCA1 and SP1, respectively. Similarly, TP53 and NKFB1 were coregulated by FOS and EGR1, respectively. Since FOS and EGR1 are linked to each other by 4 cancer-related TFs including BRCA1, SP1, TP53, and NKFB1, we also included these four TFs in the subsequent data of network construction. We screened the 4 TFs in the TRRUST version 2 database query to get each TF target genes and the DEGs from four profile datasets in GEO and TF-target genes for intersection analysis to obtain 42 differentially expressed target genes of the TFs (key genes) (Table 6), which laid the foundation for our next step to construct the gene signaling regulatory network in HCC.

**Figure 4:**
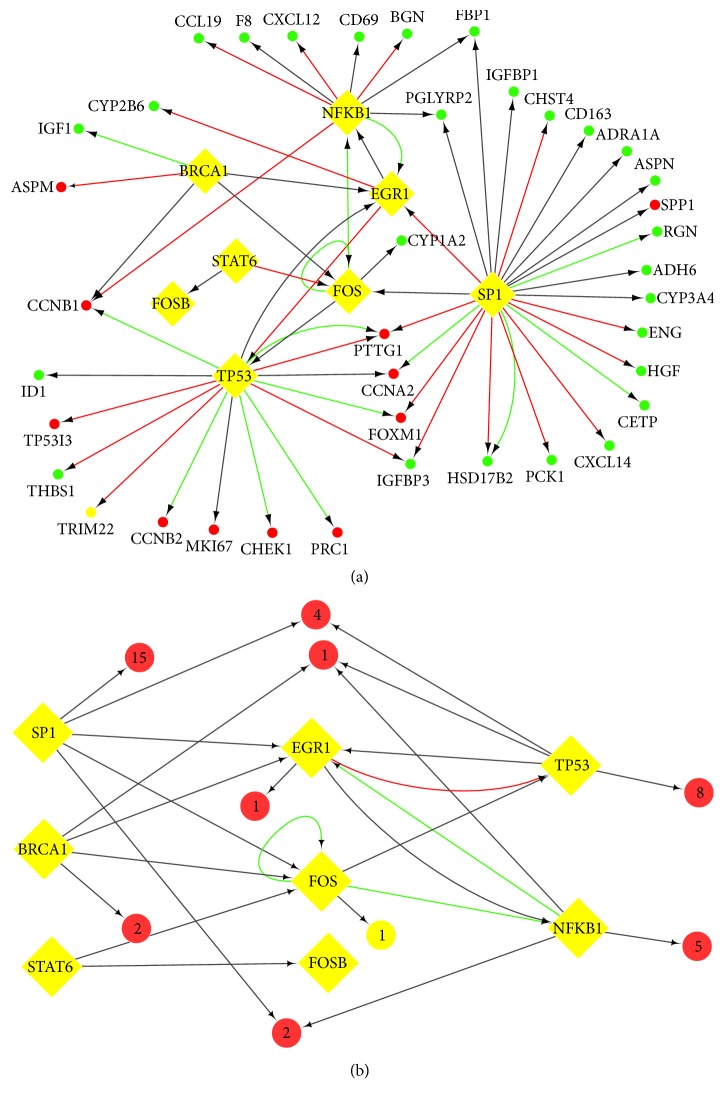
TF-related regulatory network. (a) TF-target network of these 42 key genes in HCC. (b) The brief framework of this network. TF-target network consisted of 42 nodes and 62 edges. The ellipses in the TF-gene network represented mRNAs with red (upregulated) and green (downregulated), and the diamonds represented TFs. The ellipses with a number were the clustered genes in the brief framework and the number of genes is shown inside. The interaction relationship between TFs and mRNAs were represented by arrows, and the direction of the arrow was from the source to the target. Different colors in the lines represented the different interaction relationship between the TFs and targets: red was for activation, green for repression, and grey for unknown.

Based on the above TRRUST version 2 database, we constructed the TF-target transcription regulatory network with the 42 key genes in HCC by Cytoscape 3.6.0 software (Figure 4). The TF-target network complex contained 42 key genes and 7 TFs. We performed enrichment analysis for the 42 key genes; the results were followed in Figure 6.

**Figure 5:**
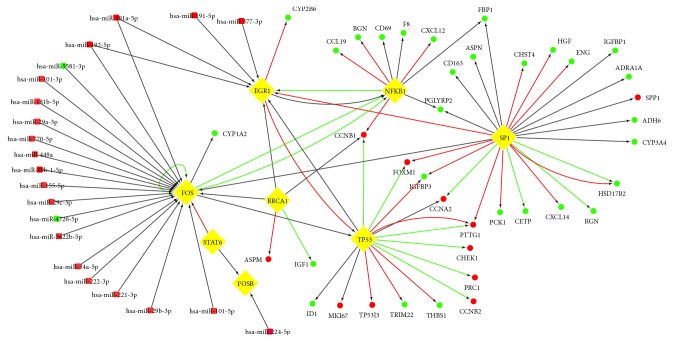
miRNA-TF-mRNA regulatory network for HCC. The squares in the network represented miRNAs, and the ellipses represented mRNAs, and the diamonds represented TFs. The nodes in red were upregulated, whereas the nodes in green were downregulated. The interaction relationship among TFs, mRNAs, and miRNAs was represented by arrows, and the direction of the arrow was from the source to the target. Different colors in the lines represented the different interaction relationship among TFs, mRNAs, and miRNAs: red was for activation, green for repression, and grey for unknown.

Subsequently, the HCC and adjacent tissue gene expression profile of GSE64632 were analyzed by GEO2R. We use p<0.05 and |logFC|>1 as cut-off criterion to obtain 703 DEMs, including 452 upregulated DEMs and 251 downregulated DEMs. The interactions between TFs and microRNAs were predicted by miRTarBase. And we performed intersection analysis between the miRNA-targeting genes and DEMs in the microRNA assay data from GEO. Based on the above TRRUST version 2 database and miRTarBase, we constructed miRNA-TF-mRNA regulatory network by Cytoscape software (Figure 5). The results showed that some genes were coregulated by the same miRNA or a few miRNAs, which suggested that miRNAs could play an important role in the progression of HCC. Using these coregulatory genes combined with regulatory networks, we constructed the miRNA-TF-mRNA regulatory network, which was shown in Figure 5. In addition, The Prediction results of differentially expressed miRNA targets revealed that EGR1 and FOS are coregulated by 2 microRNAs including hsa-miR-181a-5p and hsa-miR-192-5p, which were upregulated in HCC based on microRNA assay data. In the targeting relationship between miR-181a-5p and EGR1, FOS has been experimentally validated, especially.

### 3.3. Functional and Pathway Analysis of the Key Genes

**Figure 6:**
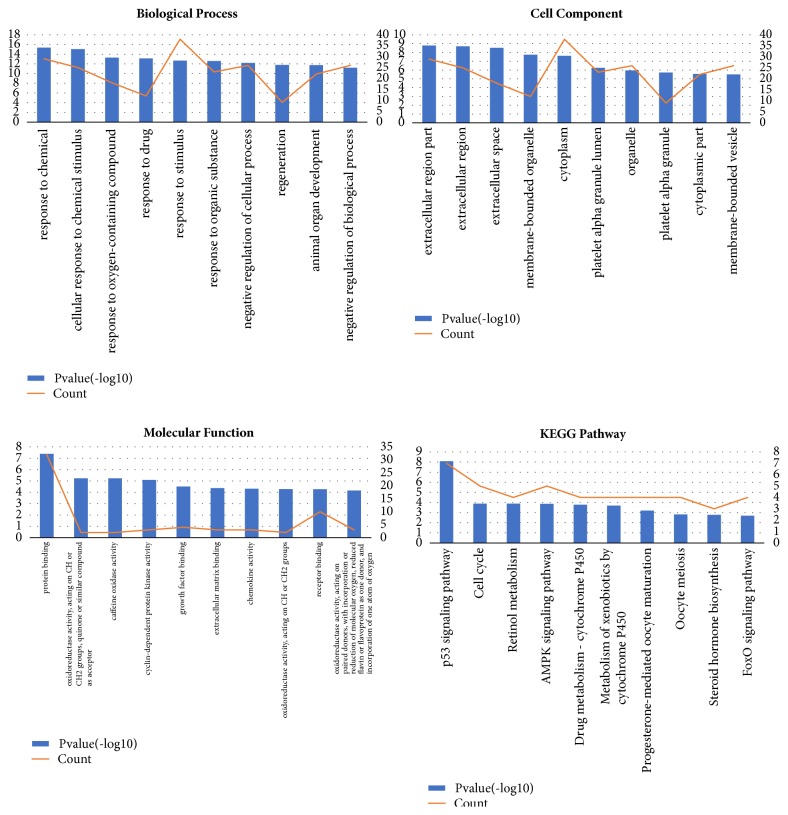
Enrichment analysis of key genes.

In the current study, we performed enrichment analysis for the 42 key genes which were differentially expressed in HCC tissues. And the results were shown in Figure 6 and Table 7. And the most enriched KEGG pathway of key genes was p53 pathway.

**Figure 7:**
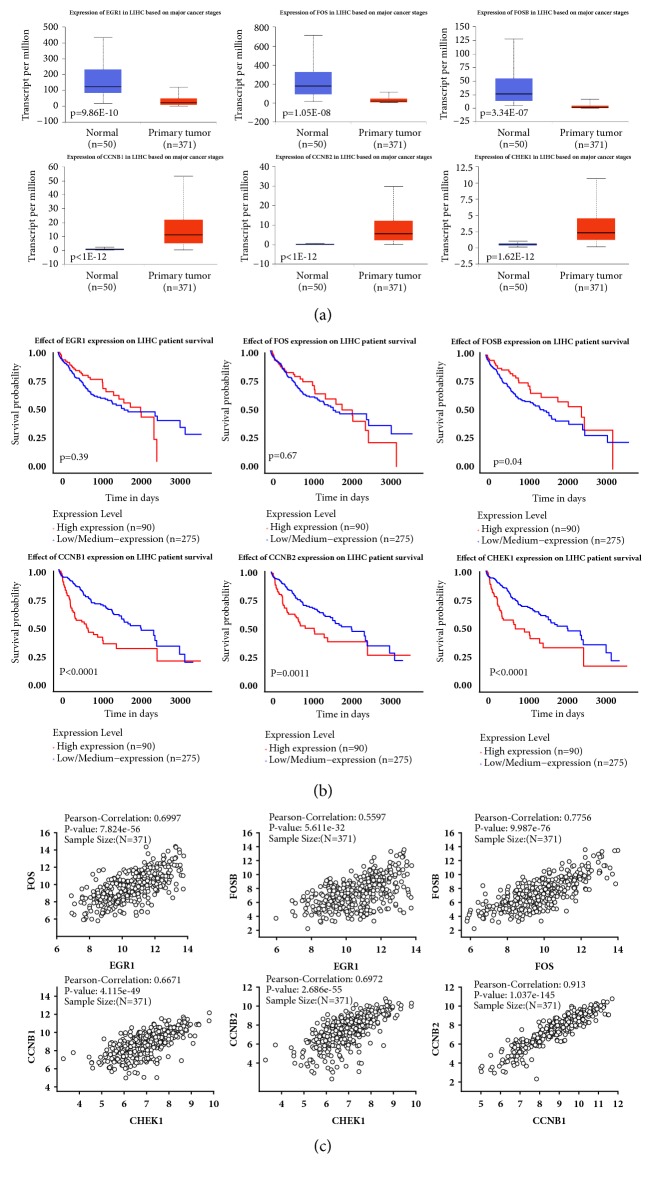
Study of the clinical association of EGR1, FOS, FOSB, CCNB1, CCNB2, and CHEK1 with the clinicopathologic parameters of hepatocellular carcinoma. (a) Boxplots depicting RNA expression levels of key genes in HCC (n = 371) versus nonmalignant liver (n = 50) from TCGA. (b) Kaplan-Meier plots comparing the overall survival rates in HCC cases (n=365) with high expression or without low/medium expression. The data was recruited from UALCAN. P<0.05 was considered statistically significant. (c) Correlation analysis of three TFs and three p53 markers. The data was recruited from Linkedomics (http://www.linkedomics.org).

We queried all the samples from TCGA liver hepatocellular carcinoma (LIHC) with RNA-seq v2 data in our study. The boxplots showed that the expression level of EGR1, FOS, and FOSB was significantly lower in primary tumor than that in the normal liver for LIHC patients from TCGA (p<0.001) (Figure 7(a)). The overall survival analysis of the three TFs was performed using the Kaplan-Meier curve. We found that the overexpression of FOSB in HCC was associated with reduced survival in cases with three TFs high expression, compared with the remaining cases with low/medium expression (Figure 7(b)). Through correlation analysis, we found that there was a strong positive correlation between the expression level of EGR1 and FOS mRNA. And there existed strong positive correlation toward coexpression of FOS and FOSB among target samples. The correlation between the expression level of EGR1 and FOSB mRNA was moderate (Figure 7(c)).

Moreover, CCNB1, CCNB2, and CHEK1, which were three of key genes in the TF-target network, were involved in the p53 pathway. The boxplots showed that the expression levels of CCNB1, CCNB2, and CHEK1 were significantly higher in primary tumor than those in the normal liver for LIHC patients from TCGA (p<0.001). The overall survival rates of patients with high expression of CCNB1, CCNB2, and CHEK1 were all significantly lower than those of patients with low/medium expression. The correlation analysis results showed that there existed strong positive correlation among CCNB1, CCNB2, and CHEK1 mRNA expression (Figure 7(c)).

### 3.4. qRT-PCR

**Figure 8:**
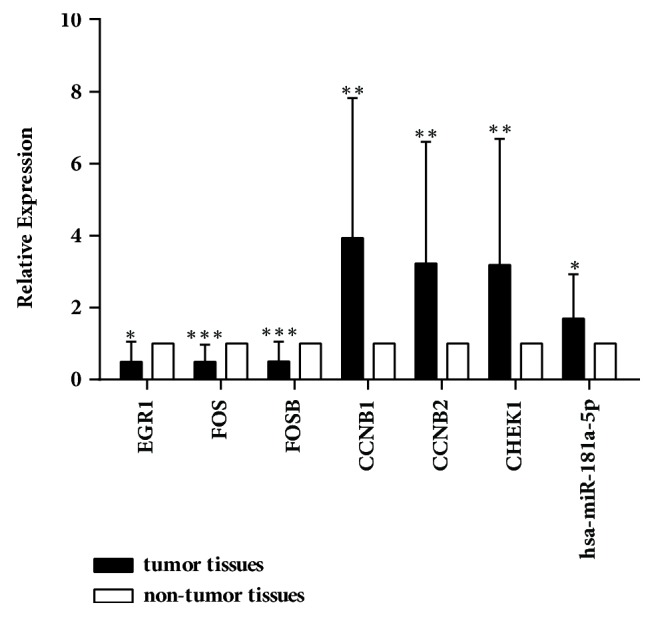
Validation of key genes and hsa-miR-181a-5p expression in 20 pairs of HCC and adjacent nontumor tissues by qRT-PCR. Detection of EGR1, FOS, FOSB, CCNB1, CCNB2, and CHEK1 mRNA expression and hsa-miR-181a-5p expression in HCC versus adjacent nontumor tissues was performed using qRT-PCR. Levels of EGR1, FOS, and FOSB mRNA were 0.493±0.558-, 0.494±0.476-, and 0.500±0.551-fold downregulated in tumor tissues, respectively, compared to those in the adjacent nontumor ones. And the levels of CCNB1, CCNB2, and CHEK1 mRNA were 3.938±3.887-, 3.225±3.388-, and 3.186±3.508-fold upregulated. The relative expression of hsa-miR-181a-5p was 1.694±1.236-fold upregulated. *∗*p<0.05, *∗∗*p<0.01, and *∗∗∗*p<0.001.

We performed qRT-PCR to examine the expression of three differentially expressed TFs including EGR1, FOS, and FOSB mRNA in HCC. The relative expressions of EGR1, FOS, and FOSB mRNA were 0.493±0.558-, 0.494±0.476-, and 0.500±0.551-fold downregulated in 20 tumor tissues versus adjacent nontumor tissues, respectively (Figure 8). Similarly, we performed qRT-PCR to explore the relationship among miR-181a-5p, TFs, and the key markers of p53 signaling pathways including CCNB1, CCNB2, and CHEK1. The relative expressions of CCNB1, CCNB2, and CHEK1 mRNA were 3.938±3.887-, 3.225±3.388-, and 3.186±3.508-fold upregulated in 20 tumor tissues versus adjacent nontumor tissues, respectively (Figure 8). And the relative expression of hsa-miR-181a-5p was 1.694±1.236-fold upregulated in 20 tumor tissues versus adjacent nontumor tissues.

## 4. Discussion

Although some progress has been made in the study of HCC, the exact mechanisms of occurrence and development in HCC are not yet clear. In the current study, we constructed networks related to transcription regulatory modes in HCC and performed functional and KEGG pathway analysis for the key genes. The enrichment analysis results showed that key genes in the TF regulatory network were enriched in the p53 signaling pathway. In addition, we performed qRT-PCR and verification in the TCGA Dataset to confirm the results based on bioinformatics analysis.

FOS (also known as c-FOS) and FOSB were both protooncogenes, which belong to the activator protein 1(AP1) family [27]. Many studies have reported that FOS was involved in proliferation, migration, and invasion of some malignancies [28, 29]. In some cases, expression of the FOS gene has also been associated with apoptotic cell death [30]. However, there still existed some contradictions about the role that c-FOS might play in the tumor progression. Some studies showed that overexpression of FOS might be associated with inhibition of tumor [31, 32]. Oliveira-Ferrer L et al. reported that c-FOS overexpression increased the apoptotic potential of ovarian cancer cells and inhibited tumor growth and metastasis, which could be achieved by changing the adhesion of human ovarian cancer cell [33]. A study by Guo J et al. showed that the expression of c-FOS in the tumor tissues of pancreatic cancer seemed to be lower than that in adjacent nontumor tissues [34]. However, some other research showed that FOS could contribute to carcinogenesis [35, 36]. Therefore, the above research work indicated that c-FOS is expressed differently in different histological types and is closely related to the proliferation, differentiation, invasion, and apoptosis of tumor cells, which provided a new therapeutic target for cancer by regulating the expression of c-FOS. EGR1 (early growth response 1) belongs to EGR family of transcription factors that includes four members: EGR1, EGR2, EGR3, and EGR4. It is a nuclear protein and functions as a transcriptional regulator, which is a component of p53 signaling [37, 38]. Abundant studies found that expression of EGR1 was associated with HCC metastasis and proliferation [39, 40]. And a number of studies suggested that EGR1 exhibits prominent tumor-suppressive activity by activating major tumor suppressor factors, including transforming growth factor-*β*1, p53, p73, fibronectin, and PTEN [40, 41]. In the present study, the mRNA expression of FOS and EGR1 was lower in HCC tissues than that in nontumor adjacent tissues based on bioinformatics analysis and qPCR verification. And the low expression of FOS and EGR1 was negatively associated with overall survival of HCC patients based on TCGA data, although this correlation was not statistically significant. The results above suggested that EGR1, FOS, and FOSB, three cancer-related TFs, could play an important role in the progression of HCC.

In the current study, we found that miR-181a-5p was upregulated in HCC based on miRNA microarray data and qRT-PCR verification. As a member of the miR-181 family, the level of miR-181a-5p was overexpressed in many cancers including breast cancers, multiple myeloma, pancreatic and gastric cancer, and hepatocellular carcinoma [42–45]. And some studies have reported that miR-181a was involved in the pathogenesis of HCC by inducing hepatocyte epithelial-mesenchymal transition and decreasing autophagy [46, 47]. A study by C. Zou et al. confirmed that miR-181a plays an important role in the progression of HCC autophagy-related modulator [48]. We found that FOS and EGR1 were both targeted by miR-181a-5p based on prediction results of key genes target, and the targeting relationship between miR-181a-5p and FOS, EGR1, has been confirmed in some studies [49, 50]. And based on correlation analysis from TCGA data, there was a tendency to a positive association between FOS and EGR1. Furthermore, the expression of FOS protein downregulated by miR-181a has been reported [51]. And another study revealed that aberrant EGR1 expression could be suppressed by miR-181a-5p directly [52].

The role of the p53 pathway in the progression of HCC has been reported in the literature [53, 54]. As an important component of the p53 pathway, TP53 (tumor protein p53) is the common target of EGR1 and FOS. Studies found that increased EGR1 expression could activate p53 signaling pathway to induce apoptosis in HCC cells [55, 56]. Association between FOS and p53 signaling has also been reported, but the specific interaction relationship is not yet clear [57]. In the current study, we found that CCNB1, CCNB2, and CHEK1 were the target genes of TP53 based on bioinformatics analysis and literature confirmation. Cyclin B1 (CCNB1) and cyclin B2 (CCNB2) are important members of the cyclin family and are important cell cycle regulators related to G2/M detection sites [58, 59]. One of its important roles is to modulate and form a complex with cyclin-dependent kinase 1 (CDK1) to phosphorylate the substrate, initiate the cell to G2/M phase from G1/S, and promote mitosis [60]. Checkpoint kinase 1 (CHEK1), as a DNA damage sensor and cell death pathway stimulator, regulates the progression of the cell cycle at the S phase, G2/M checkpoint [61]. Damaged DNA activates CHEK1, causing cell cycle arrest and repairing damaged DNA; if extensive damage is not repaired, it induces apoptosis, thereby maintaining genome integrity and stability [62]. Due to its anti-injury effect, CHEK1 plays an important role in tumor development and apoptosis [63–65]. Many studies have reported that CCNB1, CCNB2, and CHEK1 were involved in p53 signaling pathway [66–68]. We found that mRNA levels of three p53 markers including CCNB1, CCNB2, and CHEK1 were significantly upregulated in 20 HCC tumor tissues versus nontumor adjacent tissues by bioinformatics analysis and experimental verification, and their mRNA expression levels were all negatively correlated with HCC patients' survival rates (p<0.001). Therefore, we speculated that as an onco-microRNA, miR-181a-5p could inhibit expression of FOS and EGR1 to regulate p53 signaling pathway, which may be achieved through the upregulation of CCNB1, CCNB2, and CHEK1, thereby promoting the progression of HCC.

## 5. Conclusion

Using multiple cohorts profile datasets and integrated bioinformatical analysis, we identified commonly 237 DEGs, and finally found EGR1, FOS, and FOSB, 3 cancer-related TFs, which were downregulated in HCC. In addition, we constructed the transcription factor-related regulatory networks based on EGR1, FOS, and FOSB and identified possible miR-181a-5p→FOS/EGR1→p53 signaling pathway axis. It should be noted that this study examined the conclusion by qRT-PCR and bioinformatics analysis; further research needs to be done to explore more specific mechanisms. Notwithstanding its limitation, these findings significantly improved the understanding of underlying molecular mechanisms in HCC, and the key genes and pathways could be used as diagnostic and therapeutic targets and diagnostic biomarkers.

## Figures and Tables

**Table 1:** The information of expression profiles.

Series	Platform		Tissues	Adjacent tissues	Tumor tissues	Reference
GSE84402	GPL570	mRNA	Liver	14	14	[4]
GSE33006	GPL570	mRNA	Liver	3	3	[18]
GSE84005	GPL5175	mRNA	Liver	38	38	-
GSE12941	GPL5175	mRNA	Liver	10	10	[19]
GSE64632	GPL18116	miRNA	Liver	3	3	[20]

**Table 2:** The differentially expressed genes of downloaded expression profiles (P value<0.05, FC>2).

	Upregulation	Downregulation	Total
GSE84402	2232	1363	3595
GSE33006	1680	2696	4376
GSE84005	481	530	1011
GSE12941	175	385	560
Co-overexpression DEGs	57	180	237

**Table 3:** The common DEGs of four gene expression profiles (P value<0.05, FC>2).

Common DEGs	Gene symbol
Downregulated DEGs	CLEC4M, CXCL14, NAT2, FOSB, CFP, CRHBP, MARCO, CYP1A2, CLEC4G, FCN3, CNDP1, ABCA8, APOF, GYS2, GLS2, BMPER, ADRA1A, OIT3, VIPR1, GBA3, CDA, SDS, MT1G, SHBG, CYP3A4, HAMP, HAO2, GREM2, CYP39A1, HGFAC, C7, ANGPTL1, TIMD4, DPT, FOS, INMT, NNMT, TAT, C9, DNASE1L3, AKR1D1, IGJ, CYP8B1, PGLYRP2, TDO2, GPM6A, HHIP, BBOX1, CYP2B6, MT1X, RDH16, NPY1R, ZG16, DCN, GNMT, F9, C8A, HPGD, CETP, MFAP4, PLAC8, SRD5A2, AFM, ITIH4, LIFR, STAB2, HGF, C14ORF68, SRPX, TMEM27, ASPA, KLKB1, EGR1, ALDH8A1, SAA4, PHYHD1, CYP2C9, C6, DIRAS3, GHR, GNA14, ALPL, CD5L, PCK1, STEAP4, BMP5, IGF1, AGXT2, PZP, PON3, ACMSD, EPHA3, HPX, PRG4, RBMS3, ST3GAL6, FBP1, HBB, HAL, FTCD, ENO3, COLEC10, STEAP3, PROZ, CXCL12, SPP2, PDE7B, P2RY13, SLC38A4, ECM1, C8ORF4, SLC7A2, PRELP, C8B, CCL19, PTPRB, ITGA9, CHST4,ENG, RAMP3, GDF2, GPR128, ABCA9, CCDC3, VSIG4, GZMK, RND3, MBL2, LAMA2, EMR1, SOCS2, F8, CCBE1, CD69, CD163, PTGIS, FXYD1, DNAJC12, CA2, UROC1, GCH1, WDR72, RASGEF1B, COLEC11, IGFBP3, RGN, IFITM1, FBLN5, CYP2C18, CD4, GCDH, PKHD1, OLFML3, FGFR2, TEK, CPS1, TRIM22, ID1, GNAO1, SLC5A1, DHODH, PLSCR4, THBS1, ADH6, VNN1, HSD17B2, AXL, ZFP36, BGN, PDCD1LG2, SLC17A2, ASPN, SERPINA4, EHD3, FXYD6, IGFBP1, ACADSB, ADAMTS1, SFRP5, C1ORF162
Upregulated DEGs	ENAH, TXNRD1, MCM4, GNPAT, FAM38B, SORT1, SRXN1, ECT2, TARBP1, RGS5, RFX5, TKT, RAP2A, TP53I3, TUBG1, ITGA6, PRKDC, AKR1C3, CCT3, TLCD1, ATAD2, HSPB1, CENPF, NUSAP1, SLC44A5, SPP1, CHEK1, CDH13, PEG10, MKI67, ASPM, ROBO1, CCNA2, BUB1, CKAP2, CDC6, UCK2, DTL, AURKA, TPX2, MDK, DEPDC1, GPC3, CAP2, UBE2T, PTTG1, CDKN3, RRM2, PRC1, FOXM1, CCNB2, SPINK1, ANLN, KIF4A, PBK, CCNB1, GINS1

**Table:** (a) Top 10 enriched biological processes of DEGs

GO_Name	GO_ID	P value	Count
response to chemical	GO:0042221	8.60E-31	103
cellular response to chemical stimulus	GO:0070887	9.67E-27	80
response to stimulus	GO:0050896	2.83E-25	158
single-organism metabolic process	GO:0044710	1.07E-24	129
response to organic substance	GO:0010033	1.49E-22	75
small molecule metabolic process	GO:0044281	2.85E-22	76
single-multicellular organism process	GO:0044707	6.06E-20	109
negative regulation of biological process	GO:0048519	6.44E-20	91
negative regulation of cellular process	GO:0048523	8.35E-20	86
regulation of response to stimulus	GO:0048583	9.64E-20	82

**Table:** (b) Top 10 enriched cell components of DEGs

GO_Name	GO_ID	P value	Count
extracellular region	GO:0005576	1.17E-37	110
extracellular region part	GO:0044421	3.22E-35	96
membrane-bounded vesicle	GO:0031988	3.92E-29	83
extracellular space	GO:0005615	5.47E-29	60
vesicle	GO:0031982	5.72E-29	89
extracellular vesicle	GO:1903561	7.65E-27	67
extracellular organelle	GO:0043230	7.96E-27	67
extracellular membrane-bounded organelle	GO:0065010	2.19E-26	66
extracellular exosome	GO:0070062	3.55E-26	66
cytoplasmic part	GO:0044444	6.80E-14	136

**Table:** (c) Top 10 enriched molecular functions of DEGs

GO_Name	GO_ID	P value	Count
protein binding	GO:0005515	4.15E-21	146
identical protein binding	GO:0042802	2.61E-09	30
protein homodimerization activity	GO:0042803	3.74E-09	22
oxygen binding	GO:0019825	1.97E-08	8
growth factor binding	GO:0019838	4.52E-08	10
extracellular matrix binding	GO:0050840	6.88E-08	7
oxidoreductase activity	GO:0016491	7.54E-08	32
binding	GO:0005488	1.17E-07	186
protein dimerization activity	GO:0046983	1.97E-07	27
oxidoreductase activity, acting on CH or CH2 groups	GO:0016725	2.42E-07	4

**Table:** (d) Top 10 enriched KEGG pathways of DEGs

Pathway Name	Pathway ID	P value	Count
Complement and coagulation cascades	hsa04610	1.79E-06	10
Metabolic pathways	hsa01100	1.78E-05	43
p53 signaling pathway	hsa04115	3.88E-05	8
Prion diseases	hsa05020	3.97E-05	6
Histidine metabolism	hsa00340	6.78E-05	5
Retinol metabolism	hsa00830	1.93E-04	7
Steroid hormone biosynthesis	hsa00140	6.97E-04	6
Tryptophan metabolism	hsa00380	8.26E-04	5
Cell cycle	hsa04110	2.17E-03	8
Caffeine metabolism	hsa00232	3.35E-03	2

**Table 5:** Curated 36 cancer-related TF families.

Family	Full Name	Members (Official Gene Symbols)
AP1	Activator Protein 1	FOS, FOSB, JUN, JUNB, JUND

AP2	Activator Protein 2	TFAP2A, TFAP2B, TFAP2C, TFAP2D, TFAP2E

AR	Androgen Receptor	AR

ATF	Activating Transcription Factor	ATF1 – 7

BCL	B-cell CLL/lymphoma	BCL3, BCL6

BRCA	breast cancer susceptibility protein	BRCA1 – 3

CEBP	CCAAT/enhancer binding protein	CEBPA, CEBPB, CEBPD, CEBPE, CEBPG

CREB	cAMP responsive element binding protein	CREB1 - 5, CREM

E2F	E2F transcription factor	E2F1 – 7

EGR	early growth response protein	EGR1 – 4

ELK	member of ETS oncogene family	ELK1, ELK3, ELK4

ER	Estrogen Receptor	ESR1, ESR2

ERG	ets-related gene	ERG

ETS	ETS-domain transcription factor	ETS1, ETS2, ETV4, SPI1

FLI1	friend leukemia integration site1	FLI1

GLI	glioma-associated oncogene homolog	GLI1 - 4

HIF	Hypoxia-inducible factor	HIF1A, ARNT, EPAS1, HIF3A

HLF	hepatic leukemia factor	HLF

HOX	homeobox gene	HOXA, HOXB, HOXD series, CHX10, MSX1, MSX2, TLX1, PBX2

LEF	lymphoid enhancing factor	LEF1

MYB	myeloblastosis oncogene	MYB, MYBL1, MYBL2

MYC	myelocytomatosis viral oncogene homolog	MYC

NFI	nuclear factor I; CCAAT-binding transcription factor	NFIA, NFIB, NFIC, NFIX

NFKB	Nuclear factor kappa B, reticuloendotheliosis oncogene	NFKB1, NFKB2, RELA, RELB, REL

OCT	Octamer binding proteins	POU2F1 - 3, POU3F1 - 2, POU5F1

p53	P53 family	TP53, TP73L, TP73

PAX	paired box gene	PAX1 – 9

PPAR	Peroxisome proliferator-activated receptor	PPARA, PPARD, PPARG

PR	Progesterone Receptor	PGR

RAR	retinoic acid receptor	RARA, RARB, RARG

SMAD	Mothers Against Decapentaplegic homolog	SMAD1 - 9

SP	sequence-specific transcription factor	SP1 – 8

STAT	signal transducer and activator of transcription	STAT1 - 6

TAL1	T-cell acute lymphocytic leukemia-1 protein	TAL1

USF	upstream stimulatory factor	USF1, USF2

WT1	Wilms tumor 1 (zinc finger protein)	WT1

**Table 6:** Differentially expressed target genes of the transcription factor.

	Gene symbol
differentially expressed target genes of the transcription factor	CXCL14, FOSB, CYP1A2, ADRA1A, CYP3A4, FOS, PGLYRP2, CYP2B6, CETP, HGF, EGR1, PCK1, IGF1, FBP1, CXCL12, CCL19, CHST4, ENG, F8, CD69, CD163, IGFBP3, RGN, TRIM22, ID1, THBS1, ADH6, HSD17B2, BGN, ASPN, IGFBP1, CCNB1, TP53I3, PRC1, MKI67, ASPM, FOXM1, PTTG1, CHEK1, CCNB2, CCNA2, SPP1

**Table:** (a) Top 10 enriched biological processes of key genes

GO_Name	GO_ID	P value	Count
response to chemical	GO:0042221	4.35E-16	29
cellular response to chemical stimulus	GO:0070887	8.93E-16	25
response to oxygen-containing compound	GO:1901700	4.92E-14	18
response to drug	GO:0042493	7.49E-14	12
response to stimulus	GO:0050896	2.07E-13	38
response to organic substance	GO:0010033	2.53E-13	23
negative regulation of cellular process	GO:0048523	6.20E-13	26
regeneration	GO:0031099	1.64E-12	9
animal organ development	GO:0048513	1.78E-12	22
negative regulation of biological process	GO:0048519	6.23E-12	26

**Table:** (b) Top 10 enriched molecular functions of key genes

GO_Name	GO_ID	P value	Count
protein binding	GO:0005515	4.10E-08	32
oxidoreductase activity, acting on CH or CH2 groups, quinone or similar compound as acceptor	GO:0033695	5.81E-06	2
caffeine oxidase activity	GO:0034875	5.81E-06	2
cyclin-dependent protein kinase activity	GO:0097472	8.20E-06	3
growth factor binding	GO:0019838	3.10E-05	4
extracellular matrix binding	GO:0050840	4.22E-05	3
chemokine activity	GO:0008009	4.81E-05	3
oxidoreductase activity, acting on CH or CH2 groups	GO:0016725	5.31E-05	2
receptor binding	GO:0005102	5.38E-05	10
oxidoreductase activity, acting on paired donors, with incorporation or reduction of molecular oxygen, reduced flavin or flavoprotein as one donor, and incorporation of one atom of oxygen	GO:0016712	6.90E-05	3

**Table:** (c) Top 10 enriched cell components of key genes

GO_Name	GO_ID	P value	Count
extracellular region part	GO:0044421	1.59E-09	20
extracellular region	GO:0005576	1.97E-09	22
extracellular space	GO:0005615	2.94E-09	14
membrane-bounded organelle	GO:0043227	1.77E-08	38
cytoplasm	GO:0005737	2.42E-08	36
platelet alpha granule lumen	GO:0031093	5.55E-07	4
organelle	GO:0043226	1.09E-06	38
platelet alpha granule	GO:0031091	1.90E-06	4
cytoplasmic part	GO:0044444	2.72E-06	30
membrane-bounded vesicle	GO:0031988	3.14E-06	15

**Table:** (d) Top 10 enriched KEGG pathway of key genes

Pathway Name	Pathway ID	P value	Count
p53 signaling pathway	hsa04115	8.24E-09	7
Cell cycle	hsa04110	1.31E-04	5
Retinol metabolism	hsa00830	1.31E-04	4
AMPK signaling pathway	hsa04152	1.36E-04	5
Drug metabolism - cytochrome P450	hsa00982	1.65E-04	4
Metabolism of xenobiotics by cytochrome P450	hsa00980	2.06E-04	4
Progesterone-mediated oocyte maturation	hsa04914	6.36E-04	4
Oocyte meiosis	hsa04114	1.49E-03	4
Steroid hormone biosynthesis	hsa00140	1.65E-03	3
FoxO signaling pathway	hsa04068	2.04E-03	4

**Table 8:** Primer sequences used for qRT-PCR amplification.

Primer	5′ > 3′
EGR1	CACCTGACCGCAGAGTCTTT
	GAGTGGTTTGGCTGGGGTAA

FOS	GGGGCAAGGTGGAACAGTTA
	AGTTGGTCTGTCTCCGCTTG

FOSB	GCGCCGGGAACGAAATAAAC
	AACTGATCTGTCTCCGCCTG

CCNB1	AATAAGGCGAAGATCAACATGGC
	TTTGTTACCAATGTCCCCAAGAG

CCNB2	CCGACGGTGTCCAGTGATTT
	TGTTGTTTTGGTGGGTTGAACT

CHEK1	ATATGAAGCGTGCCGTAGACT
	TGCCTATGTCTGGCTCTATTCTG

GAPDH	GACAGTCAGCCGCATCTTCT
	ACCAAATCCGTTGACTCCGA

hsa-U6	CTCGCTTCGGCAGCACA
	AACGCTTCACGAATTTGCGT

hsa-miR-181a-5p	ACGCTGACCTATGAATTGACAGCC

## Data Availability

Expression profiles of GSE84402, GSE33006, GSE84005, GSE12941, and GSE64632 in the manuscript were downloaded from NCBI-GEO (https://www.ncbi.nlm.nih.gov/gds).

## Supplementary Materials

Supplementary Materials Figure S1: KEGG mapping analysis diagram of 42 key genes most enriched pathway (p53 signaling pathway). Table S1: clinicopathological characteristics of 20 HCC patients. Table S2: 62 regulation modes formed by 42 differentially expressed mRNAs in the TF-gene regulatory network. All regulation information was derived from TRRUST version 2. Table S3: prediction results of differentially expressed miRNA targets in HCC by miRTarbase.
