# Supplementary material for: Analysis of Transcription Factor-Related Regulatory Networks Based on Bioinformatics Analysis and Validation in Hepatocellular Carcinoma
Source: Biomed Res Int. 2018 Aug 29;2018:1431396. doi: 10.1155/2018/1431396 (PMC6136478; doi:10.1155/2018/1431396)

Clinicopathological characteristics of 20 HCC patients

Case	Age	Gender	Pathological Diagnosis (Differentiation Status)	HBsAg	HbeAg	HCVAb	Tumor Size(cm)	Vascular Invasion	Neural Invasion	Immunocytochemistry					
										Ki67	CK7	GPC-3	AFP	CK19	Hepatocyte
1	45	male	poorly differentiated hepatocellular carcinoma	+	+	-	5.8×5×4	-	-	30%+	-	+	+	?	-
2	37	male	poorly differentiated hepatocellular carcinoma	+	-	-	10×7×5.5	-	-	30%+	-	+	+	?	+
3	52	male	moderately differentiated hepatocellular carcinoma	+	+	-	8×6×6	+	-	10%+	Focal +	-	-	-	+
4	43	male	moderately differentiated hepatocellular carcinoma	+	-	-	3×3×2.8	-	-	10%+	Partial +	+	Focal +	Partial +	+
5	52	female	moderately/poorly differentiated hepatocellular carcinoma	+	+	-	-	-	-	Focal 5%+	-	+	Weakly +	Small focal weakly +	+
6	62	female	moderately differentiated hepatocellular carcinoma	-	-	+	4.5×4×3.5	-	-	10%+	-	-	-	?	+
7	43	male	moderately/poorly differentiated hepatocellular carcinoma	+	+	-	9×8×6	-	-	20%+	-	+	+	Focal +	-
8	53	male	moderately differentiated hepatocellular carcinoma	-	-	+	5.5×5×3	-	-	5%+	Focal +	-	-	-	+
9	65	female	moderately differentiated hepatocellular carcinoma	-	-	+	6×5×5cm	-	-	5%+	-	-	-	?	+
10	39	female	moderately/poorly	-	-	-	4×4×3.5	-	-	Focal	Small	+	+	Small	+

			differentiated hepatocellular carcinoma with necrosis							20%+	focal +			focal weakly +	
11	62	male	high/moderately differentiated hepatocellular carcinoma	+	-	-	4×3×2	-	-	Focal 10%+	-	-	-	+	
12	54	male	moderately differentiated hepatocellular carcinoma	-	-	+	-	-	-	10%+	+	?	-	-	+
13	52	male	poorly differentiated hepatocellular carcinoma	-	-	-	Diameter 2.0	+	-	20%+	-	+	-	?	+
14	16	female	high/moderately differentiated hepatocellular carcinoma	+	-	-	9×6×5.8	+	-	30%+	-	Partial +	Partial weakly +	-	Partial +
15	35	male	moderately differentiated hepatocellular carcinoma	+	-	-	8×7×6	-	-	20%+	-	-	-	?	-
16	40	male	moderately differentiated hepatocellular carcinoma	+	-	-	9×7×5.5	+	-	20%+	-	+	-	-	+
17	49	male	moderately/poorly differentiated hepatocellular carcinoma	+	-	-	9×8×6	+	-	10%+	-	+	-	-	Focal +
18	48	male	moderately differentiated hepatocellular carcinoma	+	-	-	13×8×7	-	-	30%+	-	-	-	-	-
19	52	male	poorly differentiated hepatocellular carcinoma	+	+	-	-	-	-	Focal 5%+	-	+	?	?	+
20	65	male	poorly differentiated hepatocellular carcinoma	+	+	-	-	+	-	Focal 20%+	?	+	?	-	+

Note: “?” represented for lack of relevant results; “+” represented positive results; “-” represented negative results.

Table S2 62 regulation modes formed by 42 differential genes in TF-gene regulatory network

# TF	Target	Type	Reference	# TF	Target	Type	Reference (Pubmed ID)
BRCA1	ASPM	Activation	16123590	SP1	RGN	Repression	21526343
BRCA1	CCNB1	Unknown	12647291	SP1	ADH6	Unknown	12004059
BRCA1	EGR1	Unknown	20103632	SP1	ASPN	Unknown	21528154
BRCA1	FOS	Unknown	11313879	SP1	IGFBP1	Unknown	2849945
BRCA1	IGF1	Repression	18045956	SP1	FOXM1	Activation	20372080
EGR1	CYP2B6	Activation	18303024	SP1	PTTG1	Activation	18339849
FOS	FOS	Repression	2498646	SP1	CCNA2	Repression	18368381
FOS	CYP1A2	Unknown	12135113	SP1	SPP1	Unknown	11126367
NFKB1	PGLYRP2	Unknown	16714290	SP1	HSD17B2	Activation	16807381;18270252
NFKB1	EGR1	Repression	21983014	SP1	HSD17B2	Repression	18270252
NFKB1	FBP1	Unknown	11023824	STAT6	FOSB	Unknown	18342537
NFKB1	CXCL12	Activation	17530707	STAT6	FOS	Activation	11922943
NFKB1	CCL19	Activation	20953353	TP53	CCNA2	Unknown	8270002
NFKB1	F8	Unknown	10959692	TP53	CCNB1	Repression	11892838
NFKB1	CD69	Unknown	10480634	TP53	CCNB2	Repression	12889596
NFKB1	BGN	Activation	15536164	TP53	CHEK1	Repression	15016801
NFKB1	CCNB1	Activation	11861406	TP53	EGR1	Unknown	16990849
SP1	CXCL14	Activation	22382027	TP53	FOXM1	Repression	21518729
SP1	ADRA1A	Unknown	10520459	TP53	ID1	Unknown	16557594
SP1	CYP3A4	Unknown	15100175	TP53	IGFBP3	Activation	10101800
SP1	FOS	Unknown	9528985	TP53	MKI67	Unknown	14675138
SP1	PGLYRP2	Unknown	16714290	TP53	PRC1	Repression	12889596
SP1	CETP	Repression	10669650	TP53	THBS1	Activation	10029407;10101800

SP1	HGF	Activation	9223667	TP53	TP53I3	Activation	10101800;14695213
SP1	EGR1	Activation	8349585	TP53	TRIM22	Activation	15064739
SP1	PCK1	Activation	14744869	TP53	PTTG1	Activation	12355087
SP1	FBP1	Unknown	11023824	TP53	PTTG1	Repression	15016801
SP1	CHST4	Activation	22446378	EGR1	TP53	Activation	9242687
SP1	ENG	Activation	21146604	EGR1	NFKB1	Unknown	10671503
SP1	CD163	Unknown	10725797	FOS	NFKB1	Repression	20188076
SP1	IGFBP3	Activation	12200149	FOS	TP53	Unknown	10348347

Table S3 Prediction results of differentially expressed miRNA targets in HCC by miRTarbase

miRNA	Target	miRNA	Target	miRNA	Target	miRNA	Target
hsa-miR-26a-5p	HGF	hsa-miR-194-5p	FOXM1	hsa-miR-4707-5p	CYP2B6	hsa-miR-17-5p	THBS1
hsa-miR-16-5p	HGF	hsa-miR-24-1-5p	FOXM1	hsa-miR-587	CYP2B6	hsa-miR-20a-5p	THBS1
hsa-miR-26b-5p	HGF	hsa-miR-320a	FOXM1	hsa-miR-26a-5p	PCK1	hsa-miR-92a-3p	THBS1
hsa-miR-200a-3p	HGF	hsa-miR-23a-3p	FOXM1	hsa-miR-26b-5p	PCK1	hsa-miR-155-5p	THBS1
hsa-let-7b-5p	CYP1A2	hsa-miR-204-5p	FOXM1	hsa-miR-1277-5p	PCK1	hsa-miR-30a-5p	THBS1
hsa-miR-196a-3p	CYP1A2	hsa-miR-630	FOXM1	hsa-miR-330-3p	PCK1	hsa-miR-21-5p	THBS1
hsa-miR-4252	CYP1A2	hsa-miR-423-5p	PTTG1	hsa-miR-3163	PCK1	hsa-miR-19a-3p	THBS1
hsa-miR-484	ADRA1A	hsa-miR-320a	PTTG1	hsa-miR-5582-3p	PCK1	hsa-miR-18a-5p	THBS1
hsa-miR-27b-3p	CYP3A4	hsa-miR-186-5p	PTTG1	hsa-miR-548ar-3p	PCK1	hsa-let-7d-5p	THBS1

hsa-miR-26b-5p	HSD17B2	hsa-miR-26a-5p	PTTG1	hsa-let-7f-2-3p	PCK1	hsa-let-7g-5p	THBS1
hsa-miR-4311	BGN	hsa-miR-17-5p	PTTG1	hsa-miR-26a-1-3p	PCK1	hsa-miR-27b-3p	THBS1
hsa-miR-26b-5p	ASPN	hsa-let-7b-5p	PTTG1	hsa-miR-1297	PCK1	hsa-miR-4528	THBS1
hsa-miR-29c-3p	IGFBP1	hsa-miR-146a-3p	PTTG1	hsa-miR-26a-5p	IGF1	hsa-miR-4302	THBS1
hsa-miR-1277-5p	IGFBP1	hsa-miR-26a-5p	CHEK1	hsa-miR-26b-5p	IGF1	hsa-miR-5680	THBS1
hsa-miR-625-3p	IGFBP1	hsa-miR-16-5p	CHEK1	hsa-miR-24-3p	IGF1	hsa-miR-19b-3p	THBS1
hsa-let-7b-5p	CCNB1	hsa-miR-4252	CHEK1	hsa-miR-1277-5p	IGF1	hsa-miR-4450	THBS1
hsa-miR-212-3p	CCNB1	hsa-miR-24-3p	CHEK1	hsa-miR-505-3p	IGF1	hsa-miR-1205	THBS1
hsa-miR-24-3p	CCNB1	hsa-miR-92a-3p	CHEK1	hsa-miR-29a-3p	IGF1	hsa-miR-211-3p	THBS1
hsa-miR-92a-3p	CCNB1	hsa-miR-505-3p	CHEK1	hsa-miR-455-3p	IGF1	hsa-miR-4756-5p	THBS1
hsa-miR-20a-5p	CCNB1	hsa-miR-193b-3p	CHEK1	hsa-miR-377-5p	IGF1	hsa-miR-1321	THBS1
hsa-miR-559	CCNB1	hsa-miR-424-5p	CHEK1	hsa-miR-27a-3p	IGF1	hsa-miR-3163	THBS1
hsa-miR-548b-5p	CCNB1	hsa-miR-99b-5p	CHEK1	hsa-let-7i-5p	IGF1	hsa-miR-3149	THBS1
hsa-miR-548d-5p	CCNB1	hsa-miR-15b-5p	CHEK1	hsa-miR-539-5p	IGF1	hsa-miR-5003-5p	THBS1
hsa-miR-548n	CCNB1	hsa-miR-19a-3p	CHEK1	hsa-miR-665	IGF1	hsa-miR-3065-5p	THBS1

hsa-miR-548h-5p	CCNB1	hsa-miR-19b-3p	CHEK1	hsa-miR-1305	IGF1	hsa-let-7f-5p	THBS1
hsa-miR-548i	CCNB1	hsa-miR-466	CHEK1	hsa-miR-421	IGF1	hsa-miR-4458	THBS1
hsa-miR-548w	CCNB1	hsa-miR-4672	CHEK1	hsa-miR-3065-5p	IGF1	hsa-let-7i-5p	THBS1
hsa-miR-548ab	CCNB1	hsa-miR-15a-5p	CHEK1	hsa-miR-30b-3p	IGF1	hsa-miR-4500	THBS1
hsa-miR-26b-5p	TP53I3	hsa-miR-4733-3p	CHEK1	hsa-miR-4284	IGF1	hsa-miR-455-3p	THBS1
hsa-miR-505-3p	PRC1	hsa-miR-4731-3p	CHEK1	hsa-miR-214-5p	IGF1	hsa-miR-650	THBS1
hsa-miR-125a-5p	PRC1	hsa-miR-362-3p	CHEK1	hsa-miR-4478	IGF1	hsa-miR-377-5p	THBS1
hsa-miR-2277-3p	PRC1	hsa-miR-620	CHEK1	hsa-miR-4419b	IGF1	hsa-miR-1827	THBS1
hsa-miR-302c-3p	PRC1	hsa-miR-1270	CHEK1	hsa-miR-574-5p	IGF1	hsa-miR-4537	THBS1
hsa-miR-92a-3p	PRC1	hsa-miR-4531	CHEK1	hsa-miR-18b-5p	IGF1	hsa-miR-4257	THBS1
hsa-miR-16-5p	MKI67	hsa-miR-197-3p	CHEK1	hsa-miR-28-5p	IGF1	hsa-miR-320c	THBS1
hsa-miR-484	MKI67	hsa-miR-23b-3p	CCNB2	hsa-miR-23a-3p	CXCL12	hsa-miR-192-5p	EGR1
hsa-miR-559	MKI67	hsa-let-7f-5p	CCNB2	hsa-miR-126-5p	CXCL12	hsa-miR-191-5p	EGR1
hsa-miR-548b-5p	MKI67	hsa-let-7b-5p	CCNB2	hsa-miR-126-3p	CXCL12	hsa-miR-377-3p	EGR1
hsa-miR-548d-5p	MKI67	hsa-let-7a-5p	CCNB2	hsa-miR-146a-5p	CXCL12	hsa-miR-181a-5p	EGR1

hsa-miR-548h-5p	MKI67	hsa-let-7b-5p	CCNA2	hsa-miR-1228-3p	CXCL12	hsa-miR-19b-1-5p	FOS
hsa-miR-548i	MKI67	hsa-miR-29c-3p	CCNA2	hsa-miR-362-3p	CXCL12	hsa-miR-19b-1-5p	FOS
hsa-miR-548w	MKI67	hsa-miR-27b-3p	CCNA2	hsa-miR-221-3p	CXCL12	hsa-miR-4726-5p	FOS
hsa-miR-548ab	MKI67	hsa-miR-212-3p	CCNA2	hsa-miR-454-3p	CXCL12	hsa-miR-3622b-5p	FOS
hsa-miR-505-3p	MKI67	hsa-miR-193b-3p	CCNA2	hsa-miR-148b-3p	CCL19	hsa-miR-7-5p	FOS
hsa-miR-34a-5p	MKI67	hsa-miR-29b-3p	CCNA2	hsa-miR-9-5p	CCL19	hsa-miR-146a-5p	FOS
hsa-miR-192-5p	MKI67	hsa-miR-24-3p	CCNA2	hsa-miR-26b-5p	CHST4	hsa-miR-34a-5p	FOS
hsa-miR-877-3p	MKI67	hsa-miR-19a-3p	CCNA2	hsa-let-7b-5p	ENG	hsa-miR-449a	FOS
hsa-miR-331-3p	MKI67	hsa-miR-197-3p	CCNA2	hsa-miR-1228-5p	ENG	hsa-miR-101-5p	FOS
hsa-miR-221-3p	MKI67	hsa-miR-145-5p	CCNA2	hsa-miR-3196	ENG	hsa-miR-101-3p	FOS
hsa-miR-29b-3p	MKI67	hsa-miR-148a-3p	CCNA2	hsa-miR-25-5p	ENG	hsa-miR-221-3p	FOS
hsa-miR-17-5p	MKI67	hsa-miR-148b-3p	CCNA2	hsa-miR-4781-5p	ENG	hsa-miR-222-3p	FOS
hsa-miR-218-5p	MKI67	hsa-miR-130a-3p	CCNA2	hsa-miR-32-5p	CD69	hsa-miR-181a-5p	FOS
hsa-miR-4763-5p	MKI67	hsa-miR-3666	CCNA2	hsa-miR-92a-3p	CD69	hsa-miR-29b-3p	FOS
hsa-miR-519e-3p	MKI67	hsa-miR-454-3p	CCNA2	hsa-miR-17-5p	IGFBP3	hsa-miR-192-5p	FOS

hsa-miR-5587-5p	MKI67	hsa-miR-301a-3p	CCNA2	hsa-miR-146a-5p	TRIM22	hsa-miR-155-5p	FOS
hsa-miR-92a-3p	MKI67	hsa-miR-29a-3p	CCNA2	hsa-miR-26b-5p	TRIM22	hsa-miR-181b-5p	FOS
hsa-miR-193b-3p	ASPM	hsa-miR-22-3p	CCNA2	hsa-miR-100-5p	ID1	hsa-miR-770-5p	FOS
hsa-miR-192-5p	ASPM	hsa-miR-19b-3p	CCNA2	hsa-miR-192-5p	ID1	hsa-miR-29a-3p	FOS
hsa-miR-26b-5p	ASPM	hsa-miR-455-3p	CYP2B6	hsa-miR-26b-5p	ID1	hsa-miR-29c-3p	FOS
hsa-let-7a-5p	ASPM	hsa-miR-363-5p	CYP2B6	hsa-let-7b-5p	THBS1	hsa-miR-5581-3p	FOS
hsa-miR-218-5p	ASPM	hsa-miR-377-5p	CYP2B6	hsa-miR-30a-3p	THBS1	hsa-miR-224-5p	FOSB
hsa-miR-26b-5p	FOXM1	hsa-miR-4537	CYP2B6	hsa-miR-98-5p	THBS1		
hsa-miR-186-5p	FOXM1	hsa-miR-4532	CYP2B6	hsa-let-7a-5p	THBS1		

Figure S1 KEGG mapping analysis diagram of 42 key genes most enriched pathway (p53 signaling pathway)

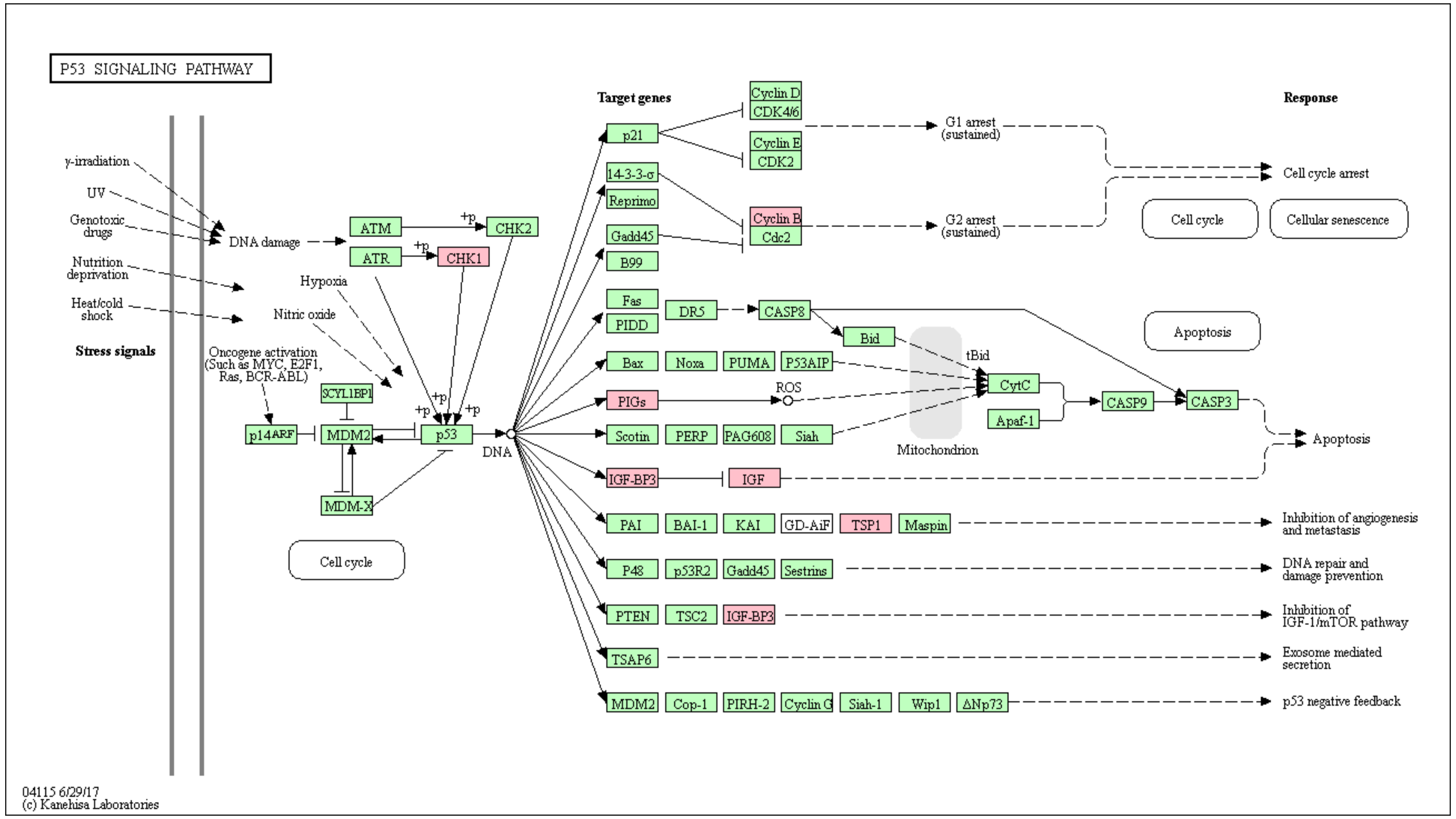

Supplement: Supplementary Materials — Figure S1: KEGG mapping analysis diagram of 42 key genes most enriched pathway (p53 signaling pathway). Table S1: clinicopathological characteristics of 20 HCC patients. Table S2: 62 regulation modes formed by 42 differentially expressed mRNAs in the TF-gene regulatory network. All regulation information was derived from TRRUST version 2. Table S3: prediction results of differentially expressed miRNA targets in HCC by miRTarbase. [file 1431396.f1.pdf]
